# Organophosphorus chemistry: from model to application

**DOI:** 10.3762/bjoc.19.8

**Published:** 2023-01-25

**Authors:** György Keglevich

**Affiliations:** 1 Department of Organic Chemistry and Technology, Budapest University of Technology and Economics, 1521 Budapest, Hungary

**Keywords:** organophosphorus chemistry

The thematic issue “Organophosphorus chemistry: from model to application” shows a number of “hot” topics of the discipline under discussion.

Cross-coupling reactions are also of importance in organophosphorus chemistry. Taddei and co-workers applied an alternative method to the classical Hirao reaction [1]. They utilized the Ni-catalyzed double Michaelis–Arbuzov reaction of bis(bromoaryl) species and related N-heterocycles to prepare the respective bisphosphonate derivatives that were transformed to the corresponding diphosphonic acids.

The Pudovik addition is still an evergreen reaction as it leads to α-hydroxyphosphonates that are versatile intermediates in organophosphorus chemistry. Yang et al. elaborated a Lewis acid-catalyzed one-pot synthesis of phosphinates and phosphonates staring from pyridinecarboxaldehydes and diarylphosphine oxides [2]. This protocol is the analogy of the Pudovik reaction, followed by the phospha-Brook rearrangement applied mainly for the synthesis of phosphoric ester derivatives.

In another study, the synthesis of bis(chlorophenyl)acetylenes that were useful for the preparation of 1,2,3-tris(chlorophenyl)cyclopropenylium bromides was accomplished [3]. The latter species were converted to tributyl(1,2,3-tris(chlorophenyl)cyclopropenyl)phosphonium bromides, affording 3,4,5-tris(chlorophenyl)-1,2-diphosphacyclopentadienides upon reaction with polyphosphides. These ligands were converted to the corresponding ferrocene complexes. This synthesis was elaborated by Zagidullin and his team.

The two remaining articles also indicate the importance of the P-heterocyclic discipline in organophosphorus chemistry.

A series of P-stereogenic chiral thiophosphorus acids, such as a fused 1-hydroxytetrahydrophosphinine 1-sulfide, an oxaphosphinine sulfide analogue, and an azaphosphinine sulfide analogue were synthesized my Montchamp and Winters as potential organocatalysts [4]. The newly prepared thiophosphorus acids were not efficient in the asymmetric transfer hydrogenation of 2-phenylquinoline. However, they may find application in other model reactions. These days, stereoselective syntheses incorporating “green" chemical considerations are of utmost importance in medicinal chemistry and beyond.

Quantum chemical calculations are a great support for organic chemists when exploring structures, reactivities, and mechanisms. In this thematic issue, the Diels–Alder cycloaddition of 2-phosphaindolizine, 1-aza-2-phosphaindolizine, 3-aza-2-phosphaindolizine, and 1,3-diaza-2-phosphaindolizine derivatives, with butadiene has been calculated by Bansal and co-workers using DFT [5]. Therein, the dienophilic reactivity of the N=P and P=C units of the heterocycles was in accordance with the experimental observations.

Additionally, a novel triferrocenyl trithiophosphite was synthesized and studied by single-crystal X-ray diffraction by Khrizanforov et al., and the preferred conformations were substantiated by DFT calculations [6].

Finally, Hersh and Chan presented a method to improve the accuracy of ^31^P NMR chemical shift calculations by use of scaling methods [7].

György Keglevich

Budapest, January 2023
